# Relapsed/refractory PBML complicated with failed platelet implantation and mixed infection after allo-HSCT: A case report

**DOI:** 10.1097/MD.0000000000050987

**Published:** 2026-10-09

**Authors:** Ying He, Fangyi Fan, Yingling Zhou, Wei Sun, Guangcui He, Yan Deng, Hai Yi

**Affiliations:** a Department of Hematology, Chinese People’s Liberation Army The General Hospital of Western Theater Command, Chengdu, Sichuan, China; b Sichuan Clinical Research Center for Hematological Disease; c Branch of National Clinical Research Center for Hematological Disease; d Department of Emergency Medicine, Chinese People’s Liberation Army The General Hospital of Western Theater Command, Chengdu, Sichuan, China; e Department of Laboratory Medicine, Chinese People’s Liberation Army The General Hospital of Western Theater Command, Chengdu, Sichuan, China.

**Keywords:** allogeneic hematopoietic stem cell transplantation, case report, mixed infection, primary bone marrow lymphoma, primary platelet engraftment failure

## Abstract

**Rationale:**

Primary bone marrow lymphoma (PBML) is a rare lymphoma with a poor prognosis, characterized by tumor origin restricted to the bone marrow. Diffuse large B-cell lymphoma (DLBCL) is its most common subtype.

**Patient concerns:**

We report a case of a 48-year-old female with refractory non-germinal center DLBCL originating in the bone marrow, accompanied by p53 overexpression, complex karyotype, and hemophagocytic lymphohistiocytosis.

**Diagnoses:**

Given the aforementioned adverse genetic features, together with primary platelet poor graft function following allogeneic hematopoietic stem cell transplantation (allo-HSCT), mixed infections, severe acute intestinal graft-versus-host disease (GVHD), and progressive bone marrow stromal necrosis, the patient was diagnosed with multifactorial post-transplant poor graft function with bone marrow microenvironment injury.

**Interventions:**

The patient received induction chemotherapy and achieved complete remission, followed by autologous-hematopoietic stem cell transplantation (autoHSCT) for consolidation. After relapse within 3 months, salvage therapy and subsequent allogeneic-hematopoietic stem cell transplantation were performed.

**Outcomes:**

Despite intensive supportive therapy, the patient died from massive gastrointestinal bleeding. A literature review identified this as a rare case of PBML with primary platelet Poor graft function and mixed infections post-allo-HSCT.

**Lessons:**

This case highlights the treatment challenges and extremely poor prognosis in ultrahigh-risk PBML with adverse genetic features and severe post-transplant complications.

## 1. Introduction

1.1 PBML is an exceedingly rare lymphoma originating in the bone marrow without evidence of lymph node, spleen, liver, or any other organ involvement.^[1,2]^ The true incidence of PBML is uncertain, as definitions have varied, but it appears to represent a distinct, aggressive type of extra nodal lymphoma.^[2–5]^ On the basis of a retrospective study of the International Extra nodal Lymphoma Study Group spanning 7 countries within 25 years, only 21 PBML cases met the criteria, reflecting its infrequency.^[1]^ Most PBMLs are B-cell non-Hodgkin lymphomas, among which DLBCLs account for the majority of PBMLs.^[2,4,6]^ DLBCLs are known for their poor prognosis, with a 2-year survival rate of 30% and a median survival of 14.9 months, and approximately 70% of patients die within 2 years despite receiving chemotherapy.^[7]^ Patients with PBML often present with B symptoms (fevers, night sweats, and weight loss) and cytopenias.^[2]^ Diagnosis requires a combination of imaging and bone marrow biopsy, which helps distinguish it from primary bone lymphoma and leukemias/lymphomas with known primary or secondary bone marrow involvement.^[1–3,8–12]^

1.2 This paper reports a case of a 48-year-old female patient who was diagnosed with DLBCL originating from the bone marrow and who presented with positive results for BCL–2, BCL–6, and MYC; p53 overexpression; a Ki-67 index as high as 90%; complex chromosomal karyotypes at the initial diagnosis; and Hemophagocytic lymphohistiocytosis (HLH). Following initial remission with R-CHOP-based chemotherapy and auto HSCT consolidation, the disease relapsed early. The subsequent clinical course after allogeneic-hematopoietic stem cell transplantation (allo-HSCT) is complicated by persistent platelet engraftment failure, a cascade of mixed infections, severe acute intestinal GVHD, and ultimately, bone marrow stromal necrosis, leading to a fatal outcome. This case illustrates the aggressive biology of certain PBML subtypes and the critical challenges in their management, particularly in the posttransplant setting.

## 2. Case report

2.1 In June 2024, a 48-year-old female patient was diagnosed with non-germinal center B-cell (Non-GCB) subtype DLBCL, complicated by HLH. There was no family history of hematological malignancies, no psychosocial disorders, and no known inherited genetic diseases. Initial laboratory work-up showed a white blood cell count of 8.49 × 10^−9^ −/L, absolute neutrophil count 5.75 × 10^−9^−/L, absolute lymphocyte count 0.86 × 10^−9^−/L, hemoglobin 64 g/L, and platelet count 119 × 10^−9^ −/L. Immature myeloid precursors were observed on peripheral blood smear, without identifiable clonal B-lymphoma cells. Bone marrow cytology demonstrated markedly hyper cellular marrow with prominent granulocytic and erythroid hyperplasia. The proportion of lymphocytes was increased, and morphologically atypical lymphocytes accounted for 21.5% of nucleated marrow cells, consistent with bone marrow infiltration by lymphoma cells. Bone marrow flow cytometry identified a population of medium-to-large mature B-cells accounting for 26.85% of total events; this neoplastic B-cell population showed partial CD5 positivity and was CD10-negative. Of note, partial CD5 expression by flow cytometry reflects weak/heterogeneous antigen expression, whereas loss of detectable CD5 signal by immunohistochemistry (IHC) likely results from lower analytical sensitivity of paraffin-based IHC for weak-partial CD5 expression. No immune phenotypic features suggestive of a precursor-type neoplasm were identified (CD34 negative; TdT was not assessed). Bone marrow biopsy demonstrated hyperplastic hematopoietic parenchyma infiltrated by numerous clusters of atypical round cells. Immunohistochemistry and in situ hybridization yielded the following results for tumor cells: CD20(+), BCL−2(+, ~80%), BCL−6(+), CD10(−), CD3(−), CD30(−), CD68(−), CyclinD1(−), MUM−1(+), MYC(+), CD34(−), CD5(−), p53(+, ~70%), Ki−67 (~(90%); ISH-EBER1/2(−). Karyotype: 49,XX,t(3;14)(q27;q32), +add(9)(q13),+18,+18[17]/46,XX[3]. Whole-body PET/CT showed no lymphadenopathy, hepatosplenomegaly, or other extra medullary lesions. Physical examination revealed no organomegaly or peripheral lymphadenopathy. The patient presented with recurrent fever, elevated transaminases, and hypertriglyceridemia, in the absence of hepatosplenomegaly or lymphadenopathy. The final diagnosis was DLBCL, Non germinal center B-cell subtype, with primary bone marrow presentation. Differential diagnoses were systematically excluded as follows: Leukemic-phase large B-cell lymphoma was excluded based on the absence of clonal neoplastic B-cells in peripheral blood on smear and flow cytometry; CD34/TdT status was used only to rule out precursor-lymphoblastic neoplasm, and was not applied for exclusion of leukemic-phase mature B-cell lymphoma. Secondary bone marrow involvement from occult extra nodal disease was excluded by negative whole-body PET/CT. and Other high-grade B-cell neoplasms involving bone marrow, including high-grade B-cell lymphoma with MYC and BCL2 rearrangements, Burkitt lymphoma, and blastoid mantle cell lymphoma, were excluded by combined evaluation of morphology, immunophenotype (CyclinD1-negative), and the absence of disease-defining genetic aberrations. As “primary bone-marrow DLBCL” is not designated as a distinct diagnostic entity in the 2022 World Health Organization (WHO)- International Consensus Classification (ICC) classification and is conceptualized as DLBCL with primary bone-marrow presentation, a high diagnostic threshold was adopted by integrating bone marrow cytology, flow cytometry, histopathology, immunohistochemistry, karyotyping, and PET/CT findings.

2.2 The patient was considered to have HLH, and dexamethasone was given for anti-inflammatory treatment. Two courses of R-ECHOP and 6 courses of R-CHOP chemotherapy were administered successively. Whole-body PET/CT revealed no abnormalities. On March 24, 2025, auto HSCT was performed. In June 2025, a whole-body PET/CT reexamination was conducted: multiple infiltrations were found in the spleen, adrenal glands, bones, etc Abnormal lymphocytes accounted for 21% of the bone marrow smear, and 22.55% monoclonal B lymphocytes were detected via bone marrow flow cytometry. The bone marrow biopsy was consistent with diffuse large B-cell lymphoma. Therapeutic effect evaluation: progression.

2.3 On July 30, 2025, after being fully informed of the patient’s condition and the next treatment options, the patient strongly requested allo-HSCT (from the younger brother to the elder sister, with HLA 9/12 matching). Pretreatment was carried out with busulfan/etoposide/fludarabine/antithymocyte globulin. Following pretransplantation conditioning, the patient experienced chemotherapy-induced bone marrow suppression, characterized by leukopenia, anemia, and thrombocytopenia. Multiple polymicrobial pathogens were detected as early as day +3 post-transplant, including *Candida krusei and Enterococcus faecalis* in sputum samples, as well as bloodstream pathogens *Enterococcus faecalis, Aspergillus flavus,* and *Aspergillus oryzae* identified by blood mNGS. After standardized combination anti-infective therapy with meropenem, teicoplanin, voriconazole, and amphotericin B, the patient’s fever resolved completely. Of note, Aspergillus oryzae is genetically closely related to Aspergillus flavus and rarely causes invasive human infection; sequence read misassignment in mNGS analysis cannot be fully ruled out. On day +6 post-transplant, granulocyte colony-stimulating factor was administered to facilitate the engraftment of white blood cells, while recombinant human thrombopoietin (rhTPO) was utilized to enhance platelet engraftment. On day +12 post-transplant, the white blood cells were active, but the platelets remained inactive (PLT < 20 × 10^9^/L) (Fig. 1). Stop using voriconazole after the white blood cell count returns to normal. The patient and her families refused to complete the chimerism. The patient also developed urinary tract infection confirmed by urine culture positive for Enterococcus faecium and hemorrhagic cystitis on day +21. Though the patient suffered transient fever and severe cough with progressive pulmonary infection on day +21, we intensified anti-infective therapy by adding voriconazole. Follow-up chest CT revealed marked alleviation of pulmonary lesions, and fever was rapidly controlled after therapeutic adjustment. Intermittent platelet infusion was administered. Owing to the limited therapeutic efficacy of platelet-raising drugs, we subsequently substituted them with rhTPO and romiplostim. Despite daily platelet transfusions and sequential use of rhTPO and romiplostim, the patient’s pre-transfusion platelet nadir remained persistently below 20 × 10^9^/L beyond day +28, meeting the diagnostic criteria for primary platelet poor graft function (PGF).^[13]^ The patient presented with recurrent nausea, vomiting, and hematochezia, and was definitively diagnosed with acute intestinal GVHD (grade III) on day +32 post-transplant. Importantly, no severe graft-versus-host disease, refractory uncontrolled infection, or myelotoxic drug exposure was observed during the day 12 to 28 evaluation window for PGF diagnosis. Grade III acute intestinal GVHD was only definitively diagnosed at day +32, which fell after the day +28 diagnostic cutoff. Although pulmonary infection aggravated on day +21, intensified voriconazole therapy achieved obvious relief of pulmonary lesions; the fever episode was rapidly resolved, and no sustained myelosuppression induced by persistent uncontrolled infection occurred within this evaluation window. Consistent with the consensus criteria described in reference,^[13]^ this case meets the definition of primary platelet-predominant PGF. A bone marrow biopsy on day +33 post-transplant indicated matrix degeneration and necrosis (Fig. 2). The patient eventually died of progressive hypotension and hypovolemic shock on day +41 post-transplant.

**Figure 1.**
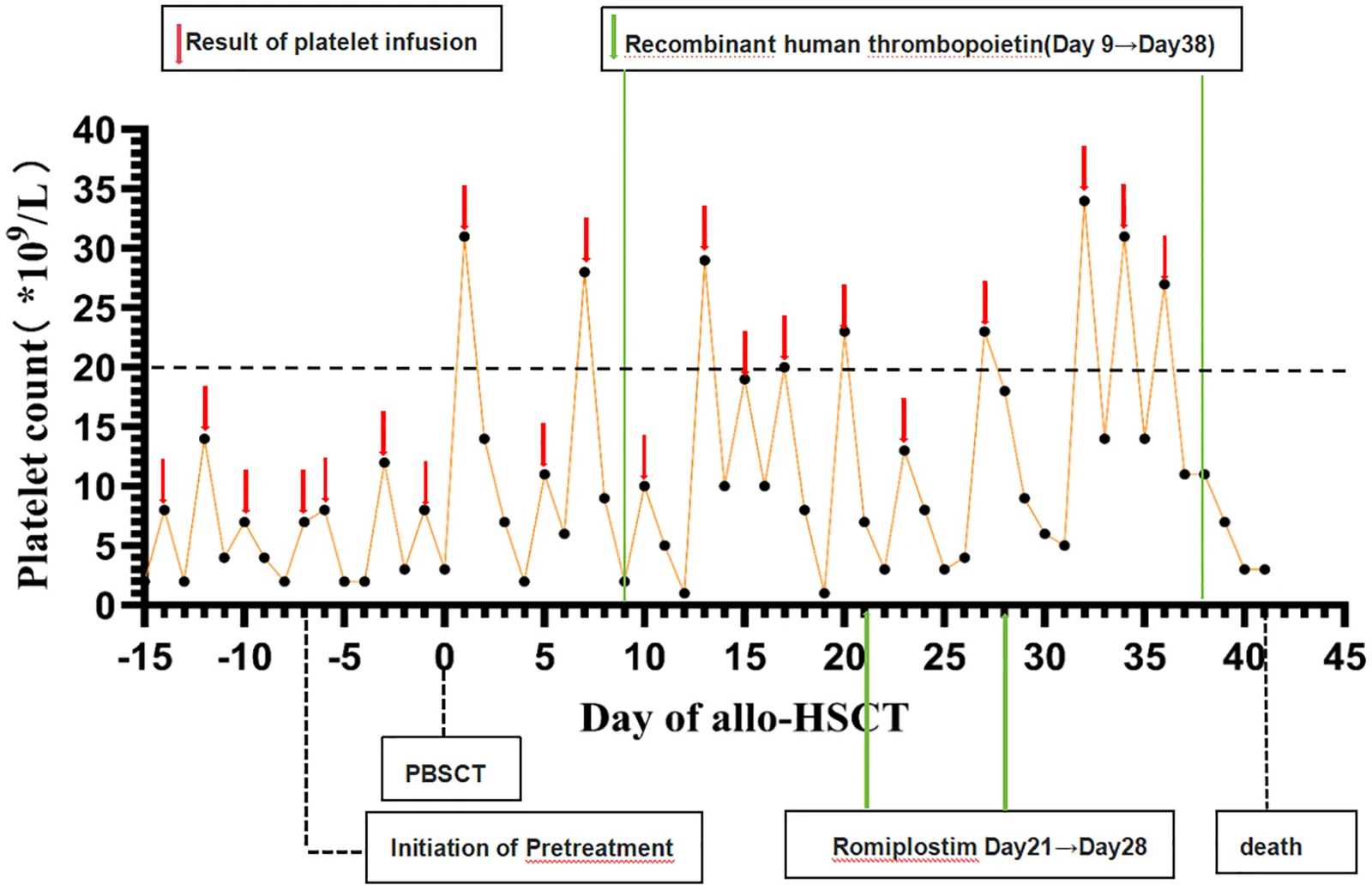
Platelet course before and after allo-HSCT. Day 0 indicates the day of stem cell infusion. The orange lines represent the trend of platelet changes throughout the entire transplantation process. The red arrow represents the corresponding platelet count after each platelet transfusion. The green arrow represents the administration periods of thrombopoietin receptor agonists: recombinant human thrombopoietin and romiplostim. The conditioning regimen (busulfan/etoposide/fludarabine/ATG) was given from Day–7 to Day–2. The horizontal black dashed line at 20 × 10^9^/L marks the threshold for platelet engraftment. Primary platelet PGF was defined as failure to achieve a sustained platelet count ≥20 × 10^9^/L without transfusion support by day + 28 post-transplant. As shown, the patient never attained sustained endogenous platelet recovery above this threshold, despite intensive platelet-raising therapy and transfusions. The patient eventually died on day + 41 post-transplant. allo-HSCT = allogeneic-hematopoietic stem cell transplantation, PGF = poor graft function.

**Figure 2.**
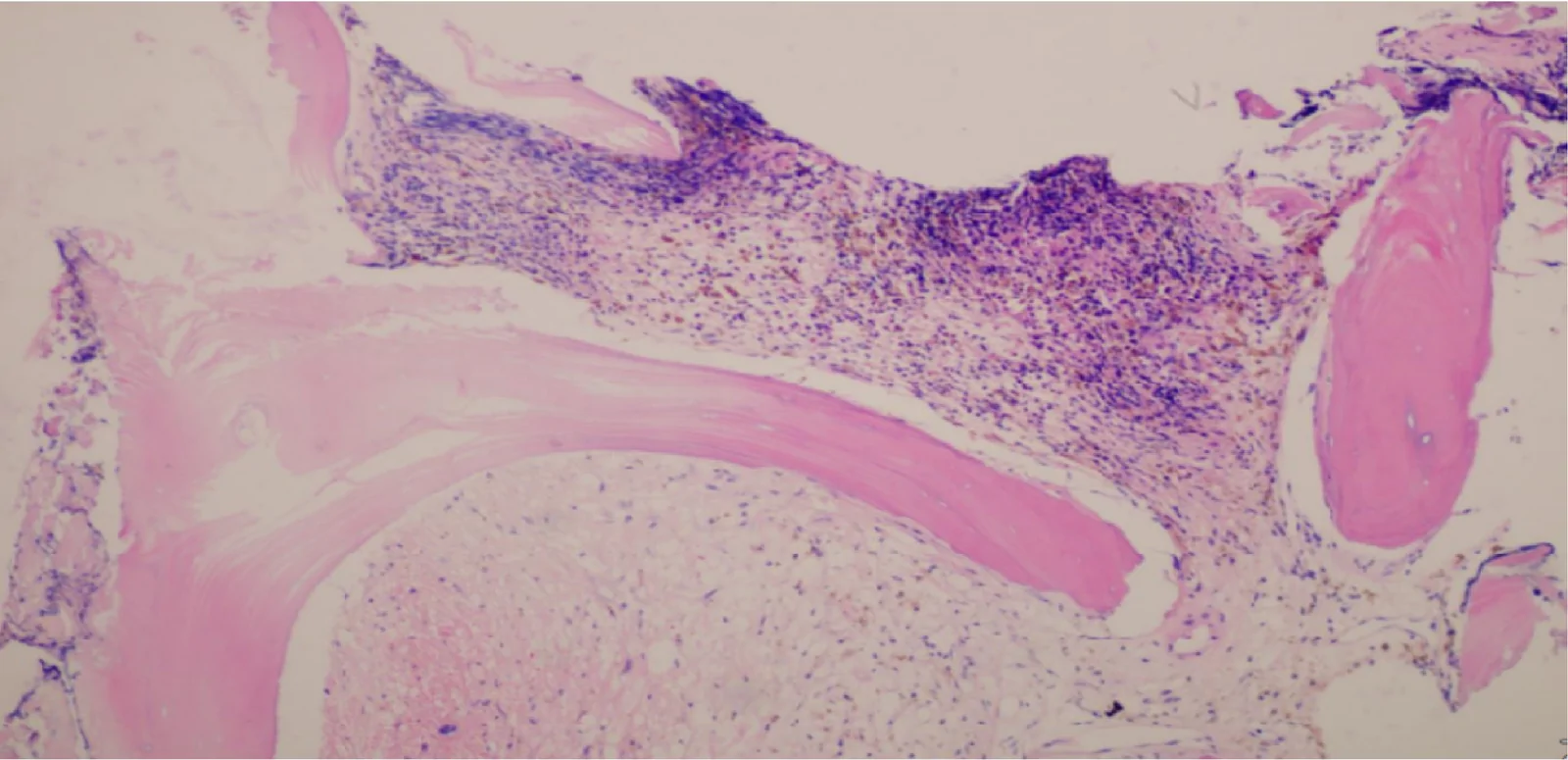
On day +33 post-transplant, the bone marrow biopsy indicated degeneration and necrosis of the bone marrow stroma.

## 3. Discussion

3.1 The present report describes one case of primary bone marrow DLBCL accompanied by primary platelet PGF and mixed infection after allo-HSCT. The diagnostic criteria for PBML have been proposed as follows: isolated bone marrow infiltration of lymphoma cells regardless of peripheral blood involvement; no evidence of lymph node, spleen, liver, or other extramedullary involvement on physical examination or imaging studies; absence of localized bone tumors; no evidence of bone trabeculae destruction in the bone marrow biopsy; and exclusion of leukemia/lymphoma cases.^[1]^ In this patient, the morphological feature of B-cell lymphoma originating from the bone marrow was CD5part + CD10- moderately large mature clonal B lymphocytes. The surface signs CD20 (+), BCL−2 (+,~ 80%), BCL−6 (+), MYC (+), and p53 (+,~70%), high expression of Ki67,~ 90%, and complex karyotypes 49, XX, t (3; 14) (q27; q32),+add(9)(q13),+18,+18[17]/46,XX[3] indicate highly aggressive B-line lymphoma. This patient met the above criteria for PBML. PBML has not yet been recognized by the ICC or the WHO classification.^[8,14,15]^ Currently, the majority of PBML cases are classified as DLBCL, not otherwise specified.^[14]^ A review indicated that 1.16% of lymphomas are PBML; however, these statistics included patients with hepatosplenomegaly, which should be excluded according to the current definition.^[4]^

3.2 A case series described a small group of patients with PBML (DLBCL subtype) whose prognosis was better than expected.^[4]^ Each patient performed well, had a good international prognostic index, and received rituximab-based treatment. In most studies, the overall survival period is very dismal. Although there is currently no recommended treatment for PBML, most studies have documented the use of R-CHOP, with a median survival of 9.6 months.^[2]^ In this case, after a clear diagnosis, the patient received 2 courses of R-ECHOP and 6 courses of R-CHOP, and the disease achieved complete remission. Subsequent auto HSCT was performed. Three months later, a whole-body PET/CT scan was conducted, revealing multiple infiltrations in the spleen, adrenal glands, and bones, among other sites. Bone marrow flow revealed 22.55% monoclonal B cells, suggesting the recurrence of lymphoma. Emergency haplo identical allo-HSCT was performed. Twelve days post-transplant, the white blood cells were active, but the platelets remained inactive (PLT < 20 × 10^9^/L) (Fig. 1). Intermittent platelet infusion was administered, and recombinant human thrombopoietin and romiplostim were successively given to increase the platelet count, but the effect was not good. Severe complications such as mixed infections at multiple sites, hemorrhagic cystitis, gastrointestinal bleeding, and acute intestinal-type GVHD (grade III) subsequently occurred. Despite treatments such as intensified anti-infectives, immunosuppressive regulation, and platelet production promotion, the condition continued to progress. On day +33 post-transplant, a reexamination of the bone marrow biopsy sample indicated matrix degeneration and necrosis. The patient eventually died due to massive gastrointestinal bleeding and hypovolemic shock.

3.3 Published retrospective series consistently demonstrate an unfavorable prognosis for PBML, with a small proportion of long-term survivors and median follow-up durations generally <24 months. In the multicenter international IELSG cohort reported by Martinez et al., 21 patients fulfilling strict diagnostic criteria for PBML were enrolled for clinicopathological analysis.^[1]^ DLBCL represented the predominant subtype of PBML in this cohort. Conventional karyotyping was successfully performed in 4 DLBCL cases; 3 of them carried highly complex karyotypes with concurrent multiple numerical and structural abnormalities, suggestive of marked genomic instability of lymphoma cells.^[1]^ Peripheral blood leukemic involvement of neoplastic B cells was rare in DLBCL, occurring in merely one of the 15 DLBCL patients, whereas 3 follicular lymphoma cases presented with circulating tumor cells.^[1]^ Kajiura et al reported that 6 out of 9 patients with PBML exhibited phagocytosis, which reflects a severe disorder of the body’s immune system.^[7]^ These scattered high-risk characteristics have been highly concentrated in this case.

3.4 This primary bone marrow lymphoma (PBML) case harbors multiple adverse high-risk biomarkers at initial diagnosis. To our knowledge, few PBML cases simultaneously exhibit such a constellation of poor prognostic features, including a triple-expressor immune phenotype defined by concurrent tumor overexpression of BCL-2, BCL-6 and MYC on IHC, p53 overexpression, an extremely high Ki-67 proliferation index of approximately 90%, and a complex karyotype. Notably, conventional karyotyping identified t(3;14)(q27;q32) consistent with BCL6 rearrangement, yet FISH testing for MYC and BCL2 gene break-apart rearrangements was not performed; per the 2022 WHO-HAEM5/ICC classification criteria, this immunophenotype cannot be classified as genetically verified double-hit or triple-hit high-grade B-cell lymphoma. This distinct adverse molecular and cytogenetic signature provides a mechanistic rationale for the patient’s highly aggressive clinical behavior and intrinsic primary chemoresistance. Although complete remission was achieved during initial chemotherapy, the patient relapsed very early after auto-HSCT, which is in line with their high-risk biological characteristics and suggests that for such patients, autologous transplantation as a consolidation treatment strategy may be insufficient. Accordingly, intensified targeted induction regimens such as polatuzumab vedotin-containing Pola-R-CHP should be prioritized at initial diagnosis for high-risk non-GCB DLBCL per POLARIX trial evidence, while chimeric antigen receptor T (CAR-T) cell therapy represents a validated salvage modality for patients experiencing post-transplant relapse.^[16–18]^ Correlational studies focusing on diffuse large B-cell lymphoma (DLBCL) consistently validate that targeted chimeric antigen receptor (CAR)-T cellular immunotherapy exerts robust anti-lymphoma cytotoxicity, yet therapeutic potency is tightly correlated with tumor surface target antigen density, mandating rigorous biomarker-based patient stratification prior to chimeric antigen receptor T infusion.^[19,20]^ At present, antigen-directed chimeric antigen receptor T regimens remain investigational rather than standard frontline or consolidation therapeutic modalities for primary bone marrow lymphoma (PBML) and high-risk DLBCL patients.^[16,21]^

3.5 Another core lesson of this case was the occurrence of fatal complications after allogeneic transplantation. Persistent primary platelet PGF meeting the criteria^[13]^ is the key to a series of subsequent vicious cycles. Studies have shown that haploid compatibility, the use of antithymocyte globulin, a previous history of intense chemotherapy, and severe GVHD are all risk factors for platelet PGF,^[13]^ and almost all of these factors were present in this patient. Primary platelet PGF not only directly leads to gastrointestinal bleeding, but the accompanying bleeding risk also limits necessary diagnostic and therapeutic procedures and seriously hinders immune reconstitution. The subsequent “waterfall-like” mixed infection and grade III acute intestinal GVHD intensified each other, forming a vicious cycle of “infection-GVHD-hemorrhage”^[22]^ and ultimately leading to irreversible bone marrow stromal necrosis.

3.6 This case highlights a critical clinical observation: for PBML carrying multiple ultrahigh-risk molecular genetic abnormalities, the success of allogeneic transplantation appears to be highly dependent on stable hematopoietic reconstitution and an intact bone marrow microenvironment. Once PGF combined with stromal necrosis occurs, the prognosis is extremely poor. This finding underscores the need for more precise risk stratification and assessment prior to transplantation in ultrahigh-risk patients. Strategies to address the potential challenges of graft failure and bone marrow recovery warrant further investigation, including optimization of bridging regimens, transplantation protocols, and post-transplant supportive care. The strength of this case report lies in its detailed documentation of a rare and fatal complication cascade (primary platelet PGF → mixed infection → GVHD → bone marrow necrosis) in an ultrahigh-risk PBML patient. However, the findings are derived from a single case and cannot be generalized. The lack of systematic literature synthesis, absence of patient-reported outcomes, and retrospective design are inherent limitations. Further prospective studies are needed to validate the observed associations.

## Acknowledgments

The authors thank the relatives of the patients for their consent in the use of their data for publication.

## Author contributions

**Conceptualization:** Ying He, Guangcui He, Yan Deng.

**Data curation:** Ying He, Yingling Zhou, Wei Sun.

**Funding acquisition:** Fangyi Fan, Hai Yi.

**Supervision:** Fangyi Fan, Hai Yi.

**Methodology:** Yingling Zhou, Wei Sun.

**Software:** Yingling Zhou, Wei Sun.

**Visualization:** Yingling Zhou, Wei Sun.

**Writing – original draft:** Ying He.

**Writing – review & editing:** Fangyi Fan, Hai Yi.
